# Relationship between age at menarche and breast cancer in individuals, as well as in first-degree kin and estrogen receptor status: a Mendelian randomization study

**DOI:** 10.3389/fonc.2024.1408132

**Published:** 2024-06-14

**Authors:** Zhijun Zhao, Jinming Zhang, Xiaofeng Tian

**Affiliations:** ^1^ Department of Thyroid and Breast Surgery, China-Japan Union Hospital of Jilin University, Jilin University, Changchun, China; ^2^ First Hospital of Jilin University, Jilin University, Changchun, China

**Keywords:** age at menarche, breast cancer, Mendelian randomization study, first-degree relatives, estrogen receptor

## Abstract

**Target:**

We executed a Mendelian randomization (MR) investigation employing two distinct cohorts of genetic instrumental variables to elucidate the causal nexus between age at menarche (AAM) and the incidence of disparate breast cancer (BC) subtypes, in addition to the incidence of BC among first-degree kin.

**Methods:**

We aggregated statistical data pertaining to AAM and BC from various consortia representing a homogenous population cohort. MR analysis was conducted employing inverse variance weighted (IVW) methodology as the principal approach, complemented by weighted median and MR-Egger regression techniques for an exhaustive evaluation. To evaluate the presence of pleiotropy, we applied the MR-Egger intercept test, MR-PRESSO, and leave-one-out sensitivity analysis.

**Results:**

Upon exclusion of confounding SNP, an increment of one standard deviation in AAM was inversely correlated with the incidence of BC. (odds ratio [OR] 0.896, 95% confidence interval [CI] 0.831–0.968)/(OR 0.998, 95% CI 0.996–0.999) and estrogen receptor-positive (ER+) BC incidence (OR 0.895, 95% CI 0.814–0.983). It was also associated with reducing the risk of maternal BC incidence (OR 0.995, 95% CI 0.990–0.999) and sibling BC incidence (OR 0.997, 95% CI 0.994–0.999). No significant association was found between AAM and estrogen receptor-negative (ER-) BC incidence (OR 0.936, 95% CI 0.845–1.037).

**Conclusion:**

Our study substantiated the causal relationship between a delayed AAM and a diminished risk of BC in probands, as well as in their maternal progenitors and siblings. Furthermore, the analysis suggests that AAM exerts a considerable potential causal influence on the risk of developing Luminal-a/b subtype of BC.

## 1 Introduction

Over recent decades, the global incidence of BC has escalated, positioning it as a principal threat to women’s health and a leading cause of cancer-related mortality among women worldwide (1). The global prevalence of BC is escalating at an approximate rate of 2.0% annually (2, 3). BC, a malignant neoplasm originating in the breast’s epithelial tissue, predominantly affects females. The aberrant cells often lack typical cellular characteristics, readily disassociate from the primary tumor, disseminate via blood or lymphatic vessels, and metastasize, thereby posing a substantial threat to patients’ survival. The etiology of BC is multifactorial, with emerging research suggesting that the interplay between endogenous genetic factors and exogenous environmental factors may underpin the rising incidence of BC.

The AAM signifies a critical juncture in female development, marking the commencement of ovarian and reproductive-related endocrine activities instigated by the reactivation of the hypothalamic-pituitary-gonadal (HPG) axis. AAM is modulated by both environmental and genetic factors (4). Over the past century, AAM has been progressively declining in most developed and developing nations (5). Existing literature suggests that factors such as body mass index (BMI), birth weight, adolescent height, socioeconomic status, obesity, and educational level can concurrently influence AAM and BC risk.

Previous investigations have probed the correlation between AAM and BC incidence, indicating that for every one-year decrease in AAM, the risk of BC escalates by 1.050 times (1.050, 95% CI: 1.044–1.057, P<0.0001) (6). However, some researchers contend that the association between AAM and BC is not statistically significant (5). The majority of these studies are epidemiological and case-control in nature, which inherently limits their ability to establish causality. Furthermore, observational studies may not adequately control for confounding risk factors, potentially compromising the validity of their conclusions. MR is a methodological approach that investigates potential causal associations between an exposure and an outcome, utilizing genetic variants as instrumental variables. These genetic variants are randomly assigned to progeny during the course of genetic inheritance, thereby rendering them impervious to conventional confounding variables and fulfilling the prerequisite of temporal precedence. Consequently, MR is capable of surmounting certain constraints inherent in traditional observational epidemiology (7).

Consequently, this study aims to elucidate the causal relationship between AAM (exposure) and BC (outcome) utilizing MR analysis. This analysis will be based on genome-wide association studies (GWAS) data derived from European populations.

## 2 Methods

**Figure 1:**
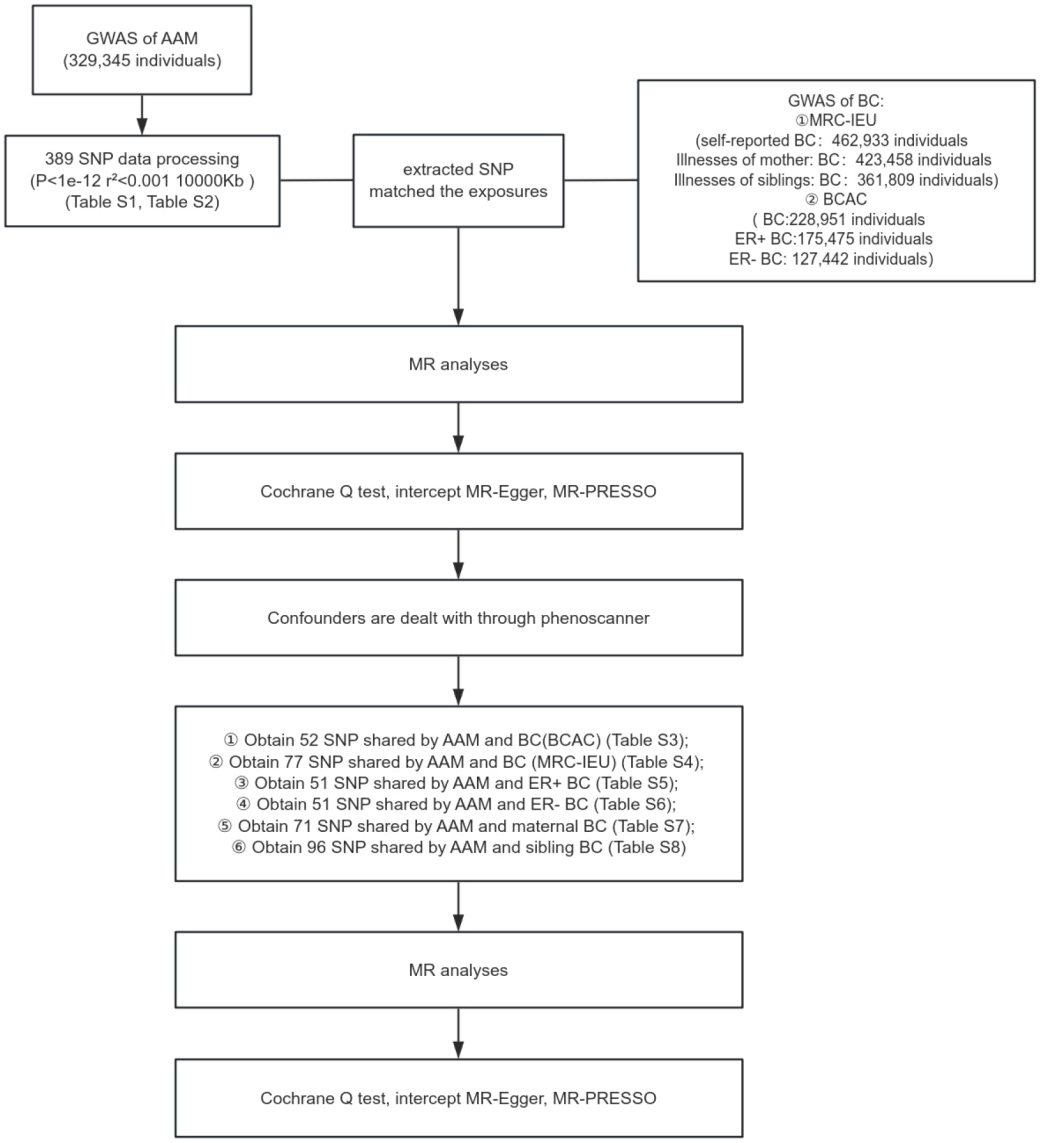
Schematic representation of MR study delineating the causal inference from AAM to BC incidence.

In the present investigation, a two-sample MR framework was adopted, leveraging data derived from discrete and non-overlapping GWAS. The employment of this methodology augments the robustness of the research findings and attenuates concerns pertaining to sample overlap that could potentially confound the results. The architectural blueprint of the study design is delineated in **Figure 1**. Given the retrospective nature of the analysis, which is predicated on the examination of data from prior publications, the study is exempt from the requisites of ethical committee approval and the procurement of informed consent.

Research design of this study. GWAS data of AAM was obtianed from the ReproGen consortium, 23andMe, and UK Biobank as exposure data, the MRC-IEU consortium obtained GWAS data of BC(MRC-IEU) and BC(siblings) and BC(mother) and the BCAC consortium obtained GWAS data of ER+ BC and ER- BC and BC(BCAC) as outcome data to analyze the causal relationship.

### 2.1 GWAS data for AAM

GWAS data pertaining to AAM in European females were procured from an integrated analysis conducted by three consortia, as detailed on the ReproGen Consortium website (https://www.reprogen.org/), encompassing a cohort of 329,345 participants. Felix R Day et al. identified 389 genetic signals associated with AAM (8) (**Supplementary Table S1**).

### 2.2 Selection of instrumental variables

To satisfy the assumption of association, genetic variants with a significance level of *p*< 1×10^-12^ and an F-statistic exceeding the conventional value of 10 were selected. These criteria were used to identify robust instrumental variables and exclude weak ones. To meet the assumption of independence, a filtering condition of r^2^ ≤ 0.001 and Kb=10,000 was implemented, resulting in 102 single-nucleotide polymorphism (SNP) markers strongly associated with AAM (9). Additional details on the selected SNP markers are provided in **Supplementary Table S2**. We extracted SNP associated with the exposure variable, AAM, from the BC outcome dataset. These SNP were then processed through the Phenotype Scanner (http://www.phenoscanner.medschl.cam.ac.uk/phenoscanner) to control for potential confounding variables. We identified 52 SNP that were common to both AAM and BC as per the Breast Cancer Association Consortium (BCAC) data, presented in **Supplementary Table S3**. Furthermore, we found 77 overlapping SNP between AAM and BC according to the MRC-IEU dataset, as shown in **Supplementary Table S4**. In **Supplementary Table S5**, we report 51 SNP that are shared between AAM and ER+ BC, while **Supplementary Table S6** lists 51 SNP common to AAM and ER- BC. Additionally, **Supplementary Table S7** details 71 SNP that are shared between AAM and maternal BC, and **Supplementary Table S8** enumerates 96 SNP that are common to AAM and sibling BC.

### 2.3 GWAS data for breast cancer

The genotypic information pertinent to breast carcinoma was procured from GWAS database, accessible via the URL: http://gwas.mrcieu.ac.uk. This dataset encompasses patient-derived data, aggregating to a comprehensive cohort of 462,933 specimens ascertained by MRC-IEU, in conjunction with an additional compilation of 228,951 specimens curated by BCAC (10). Data for mothers and siblings with BC were obtained from 423,458 and 361,809 samples from the MRC-IEU, respectively. Additionally, data pertaining to ER+ and ER- BC were procured from 175,475 and 127,442 samples from BCAC, respectively (11). All GWAS population datasets employed in the MR analysis were derived from individuals of European descent, a measure taken to mitigate potential confounding biases attributable to population stratification. Comprehensive details of the GWAS cohorts included are delineated in **Table 1**.

**Table 1:** Source of all GWAS.

Consortium	Phenotype	Sample size	Web source
A meta-analysis of GWAS	AAM	329,345	https://www.gen.org/
MRC-IEU	BC	462,933	https://gwas.mrcieu.ac.uk/datasets/ukb-b-16890/
MRC-IEU	mother: BC	423,458	https://gwas.mrcieu.ac.uk/datasets/ukb-b-13584/
MRC-IEU	siblings: BC	361,809	https://gwas.mrcieu.ac.uk/datasets/ukb-b-12227/
BCAC	BC	228,951	https://gwas.mrcieu.ac.uk/datasets/ieu-a-1126/
BCAC	ER+ BC	175,475	https://gwas.mrcieu.ac.uk/datasets/ieu-a-1127/
BCAC	ER-BC	127,442	https://gwas.mrcieu.ac.uk/datasets/ieu-a-1128/

#### 2.4.1 MR analyses

IVW Method: This is the principal analysis method used. It systematically assigns weights to each SNP-derived estimate reflecting the effect of AAM on BC risk, based on the reciprocal of its variance. Subsequently, these weighted estimates are amalgamated to yield a composite effect size. This technique is amenable to application within the framework of either a fixed-effects or a random-effects model, contingent upon the underlying assumptions regarding between-study heterogeneity.

MR-Egger Regression: The MR-Egger regression is delineated as an ancillary analytical tool supplementary to the IVW method. Its utility lies in the detection and adjustment for potential pleiotropic effects, wherein the genetic instrument may influence the outcome via mechanisms that are independent of the exposure under investigation. The MR-Egger regression generates an intercept term, the statistical significance of which may be indicative of pleiotropic bias within the causal inference framework.

Weighted Median Method: Employed as an additional supplementary technique, the Weighted Median method offers a robust estimation of the causal effect by computing the median of the SNP-weighted estimates. This approach demonstrates increased resilience to infractions of the instrumental variable assumptions, thereby enhancing the reliability of the causal inference.

In the context of MR studies, these methods are used to strengthen the analysis and provide more reliable causal estimates.

#### 2.4.2 Sensitivity analyses

The Cochran’s Q test is employed to evaluate heterogeneity across the single effect size estimates derived from instrumental variables. P<0.05 resulting from Cochran’s Q test indicates significant heterogeneity among these estimates. Within the framework of MR analysis, such heterogeneity may suggest that the instrumental variables do not uniformly estimate the same causal effect. Upon detection of heterogeneity, MR analysis typically resorts to a random-effects model to accommodate the observed variability.

MR-Egger regression is utilized to detect and adjust for horizontal pleiotropy, which arises when instrumental variables influence the outcome via pathways that do not involve the exposure of interest. The intercept term within the MR-Egger regression serves as a diagnostic tool. P-value for the intercept less than 0.05 implies the existence of pleiotropy.

The”leave-one-out” sensitivity test constitutes an essential approach in MR investigations, aiming to ascertain the robustness of the findings and to pinpoint any individual SNP that may exert undue influence on the causal estimates. This technique is instrumental in evaluating the dependability of MR results and in identifying potential sources of bias or confounding.

MR-PRESSO is an invaluable instrument for the identification and rectification of horizontal pleiotropy within Mendelian randomization studies. It facilitates the detection and correction of potential violations of the assumptions underpinning MR, thereby augmenting the credibility of causal inferences in the field of genetic epidemiology.

## 3 Results

### 3.1 The AAM was related to BC

**Figure 2:**
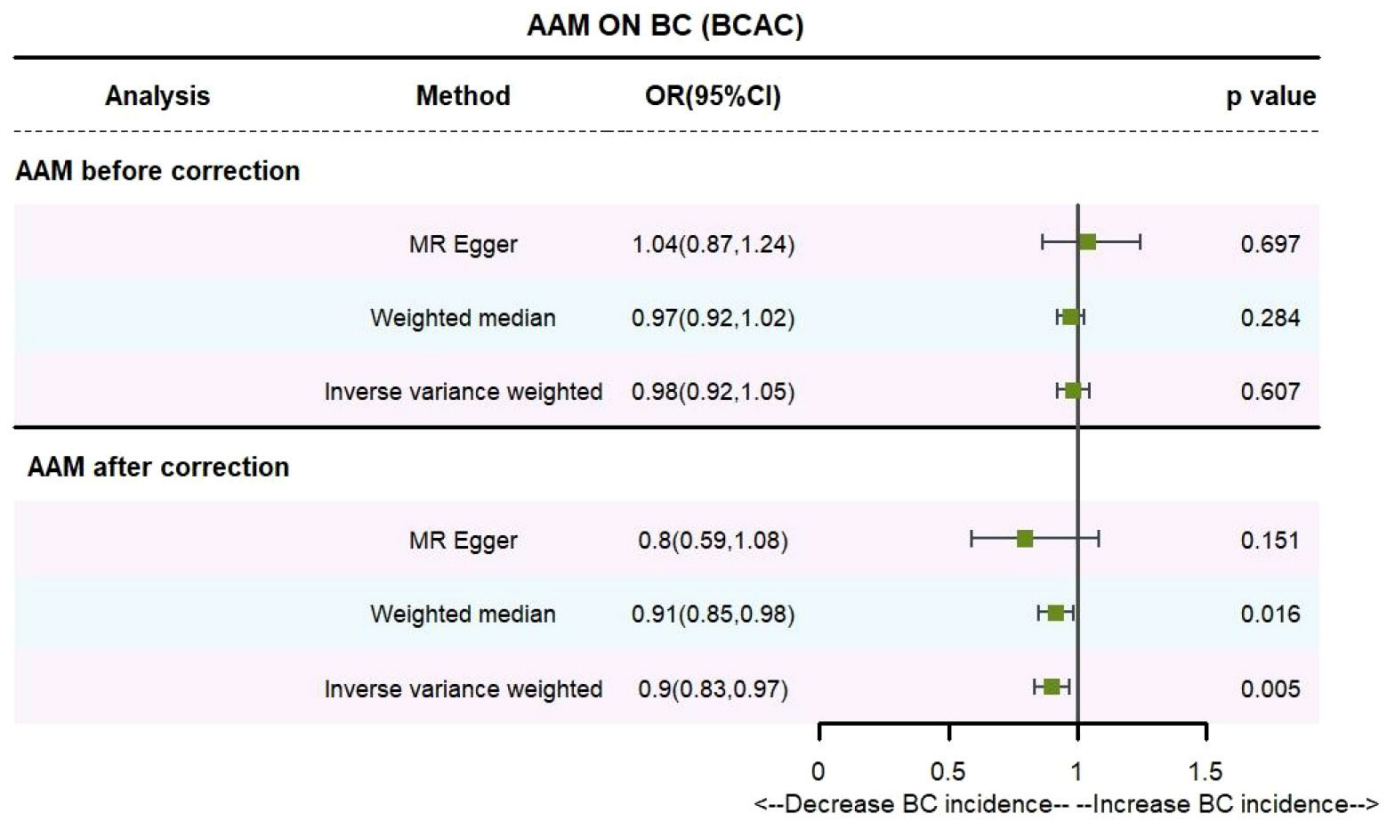
Causality of AAM with BC (BCAC) OR chart for AAM and BC (BCAC).

**Figure 3:**
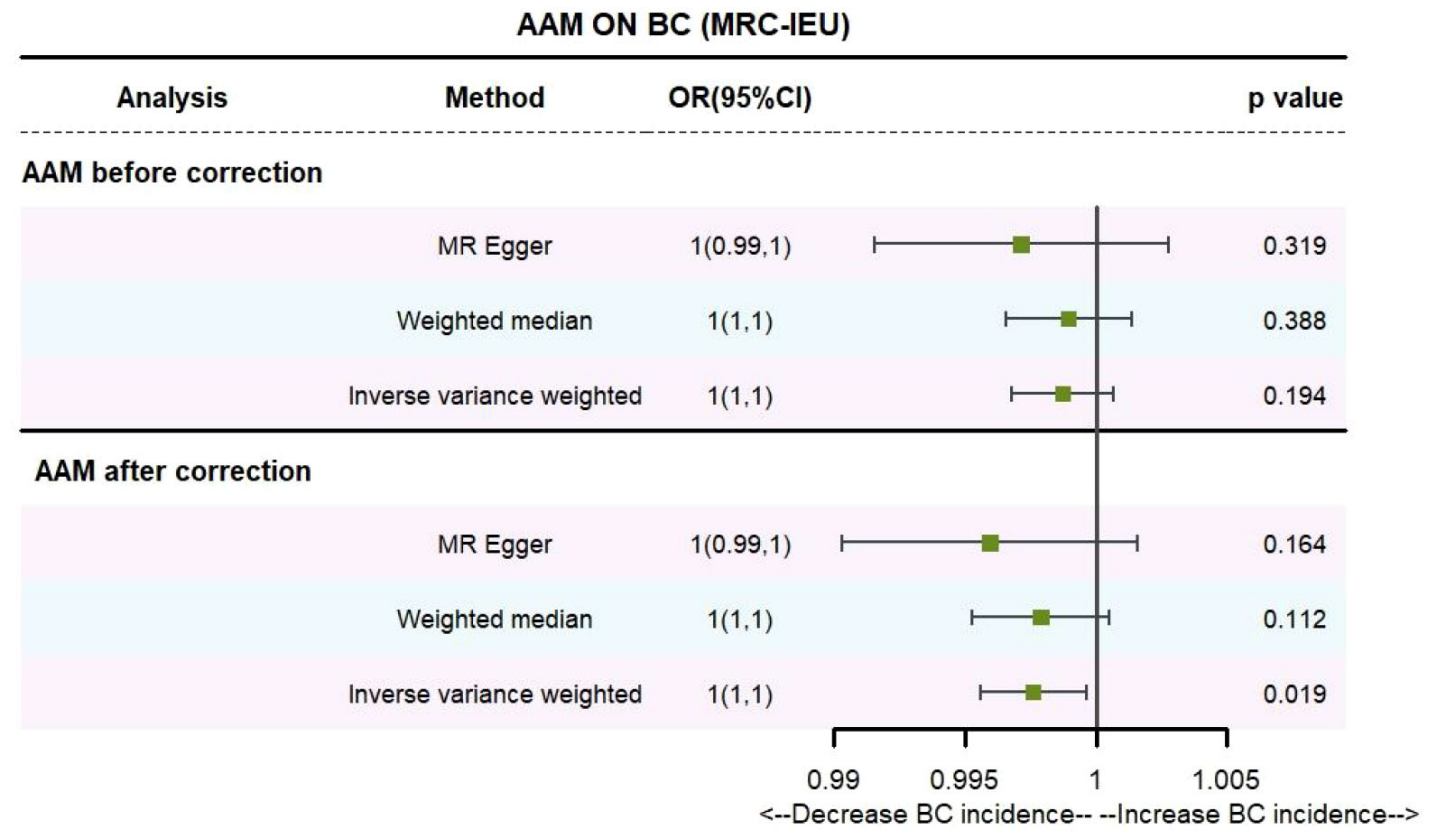
Causality of AAM with BC (MRC-IEU) OR chart for AAM and BC (MRC-IEU).

Utilizing IVW method, subsequent to the adjustment for confounding variables within the Phenoscanner database, the adjusted effect sizes for AAM on BC within the BRAC dataset were observed to be (OR, 0.896, [95% CI, 0.831–0.968], P=0.005, **Figure 2**), and within the MRC-IEU dataset, the effect sizes were (OR, 0.998, [95% CI, 0.996–0.999], P=0.019, **Figure 3**).

Employing the MR-Egger regression analysis, the effect sizes for BC within the BRAC dataset were quantified as (OR, 0.796, [95% CI, 0.587–1.080], P=0.151, **Figure 2**), and within the MRC-IEU dataset, the effect sizes were quantified as (OR, 0.996, [95% CI, 0.990–1.002], P=0.164, **Figure 3**). Furthermore, the weighted median estimation yielded effect sizes for BC within the BRAC dataset as (OR, 0.913, [95% CI, 0.848–0.983], P=0.016, **Figure 2**), and within the MRC-IEU dataset as (OR, 0.998, [95% CI, 0.995–1.000], P=0.112, **Figure 3**).

Collectively, these results imply that later AAM exerts a significant potential causal influence and constitutes a reduced risk for the development of BC across diverse datasets.

#### 3.1.1 The Sensitivity analyses of causality of AAM with BC

**Figure 4:**
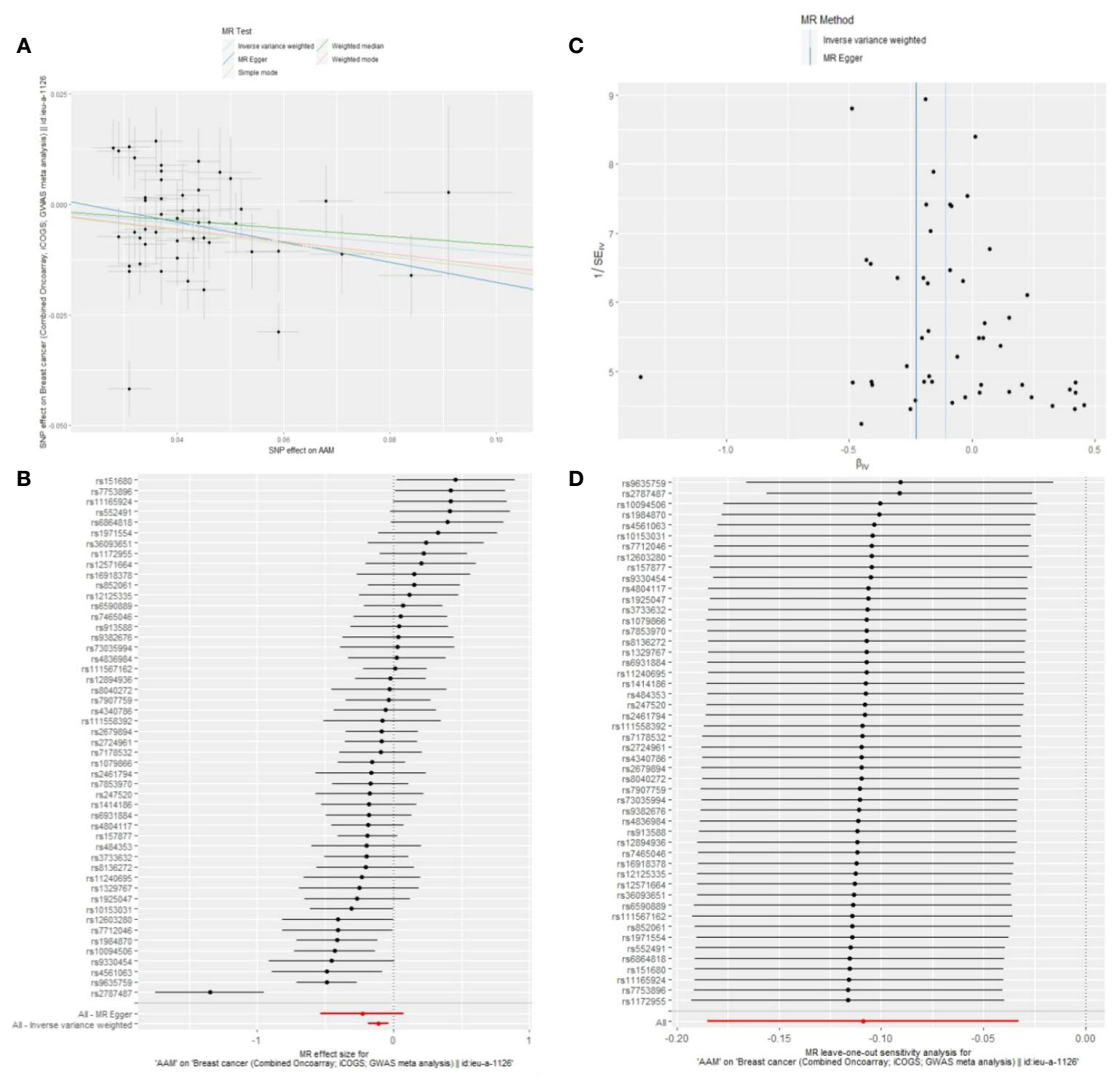
Summary figures of causality of AAM with BC (BCAC) (MRC-IEU). **(A)** Scatter Plot. **(B)** Forest Plot. **(C)** Funnel Plot. **(D)** Leave-One-Out Plot. The scatter plot demonstrates a negative correlation between AAM and BC. Leave-one-out analysis indicates that the comprehensive effect remains unchanged or reverses after the removal of any SNP, suggesting the reliability of the results. This supports the presence of a negative correlation between the AAM and BC. Forest Plot demonstrates a negative correlation between AAM and BC. Funnel Plot exhibits a symmetrical pattern.

**Figure 5:**
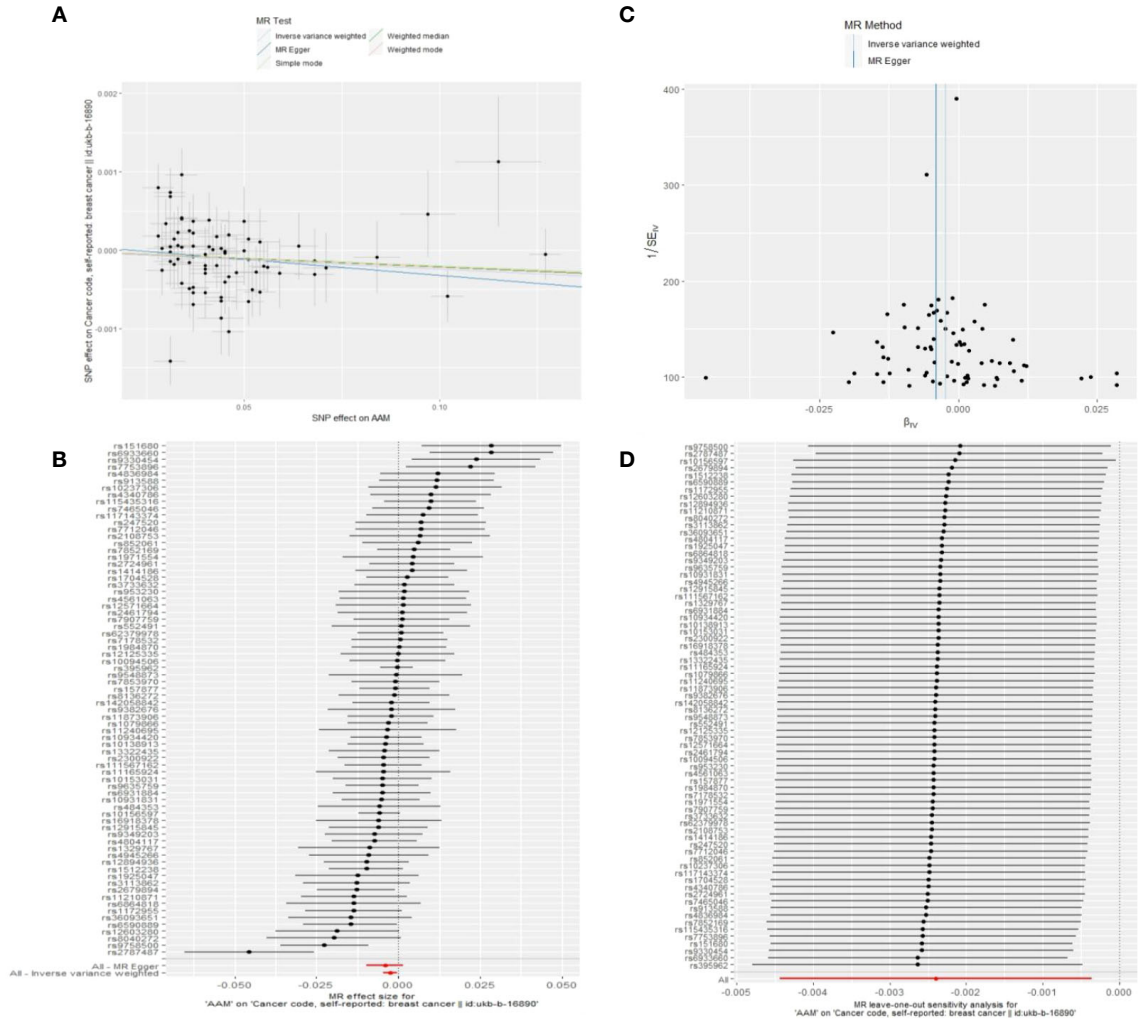
Summary figures of causality of AAM with BC. **(A)** Scatter Plot. **(B)** Forest Plot. **(C)** Funnel Plot. **(D)** Leave-One-Out Plot. The scatter plot demonstrates a negative correlation between AAM and BC. Leave-one-out analysis indicates that the comprehensive effect remains unchanged or reverses after the removal of any SNP, suggesting the reliability of the results. This supports the presence of a negative correlation between the AAM and BC. Forest Plot demonstrates a negative correlation between AAM and BC. Funnel Plot exhibits a symmetrical pattern.

We executed a Cochran’s Q test to evaluate a presence of heterogeneity within our analysis: for BC (BRCA), the inferential statistics yielded an IVW Q_pval=7.876249e-09, and for BC (MRC-IEU), an IVW Q_pval=0.003. Additionally, we conducted an analysis for potential pleiotropic effects: for BC (BRCA), the egger_intercept P=0.434, and for BC (MRC-IEU), the egger_intercept P=0.538. In MR-PRESSO framework, the overall test provided a result of P<0.001 for AAM and BC (BRCA), and a result of P=0.003 for BC (MRC-IEU). These findings suggest an absence of heterogeneity and outlier effects. The data robustly support a deterministic association between AAM and BC. Our “leave-one-out” sensitivity analysis displayed that no SNP created a substantial effect on the overall results, thereby confirming the reliability of our findings. The funnel plot analysis indicated that the potential for heterogeneity bias within our study was statistically non-significant and exhibited a symmetrical distribution, as depicted in (**Figures 4**, **5**) (**Table 2**).

**Table 2:** Sensitivity analysis of AAM and BC.

Exposure	Outcome	Heterogeneity Q p-value	MR-Egger regression Egger intercept	MR-PRESSO Global test p-value
AAM	BC (BRCA)	7.876249e-09	0.434	<0.001
AAM	BC (MRC-IEU)	0.003	0.538	0.003
AAM	ER+ BC	3.476167e-09	0.278	<0.001
AAM	ER- BC	0.059	0.342	0.041
AAM	maternal BC	0.003	0.736	0.004
AAM	sibling BC	0.043	0.375	0.026

AAM, age at menarche; BC, Breast Cancer; ER+, estrogen receptor-positive; ER-, estrogen receptor-negative.

### 3.2 The AAM was related to Luminal-a/b BC

**Figure 6:**
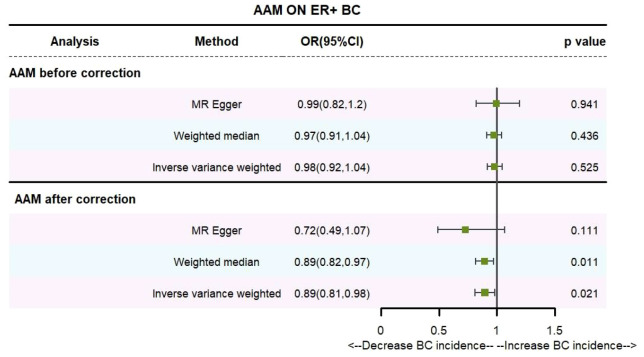
Causality of AAM with ER+ BC OR chart for AAM and ER+ BC.

**Figure 7:**
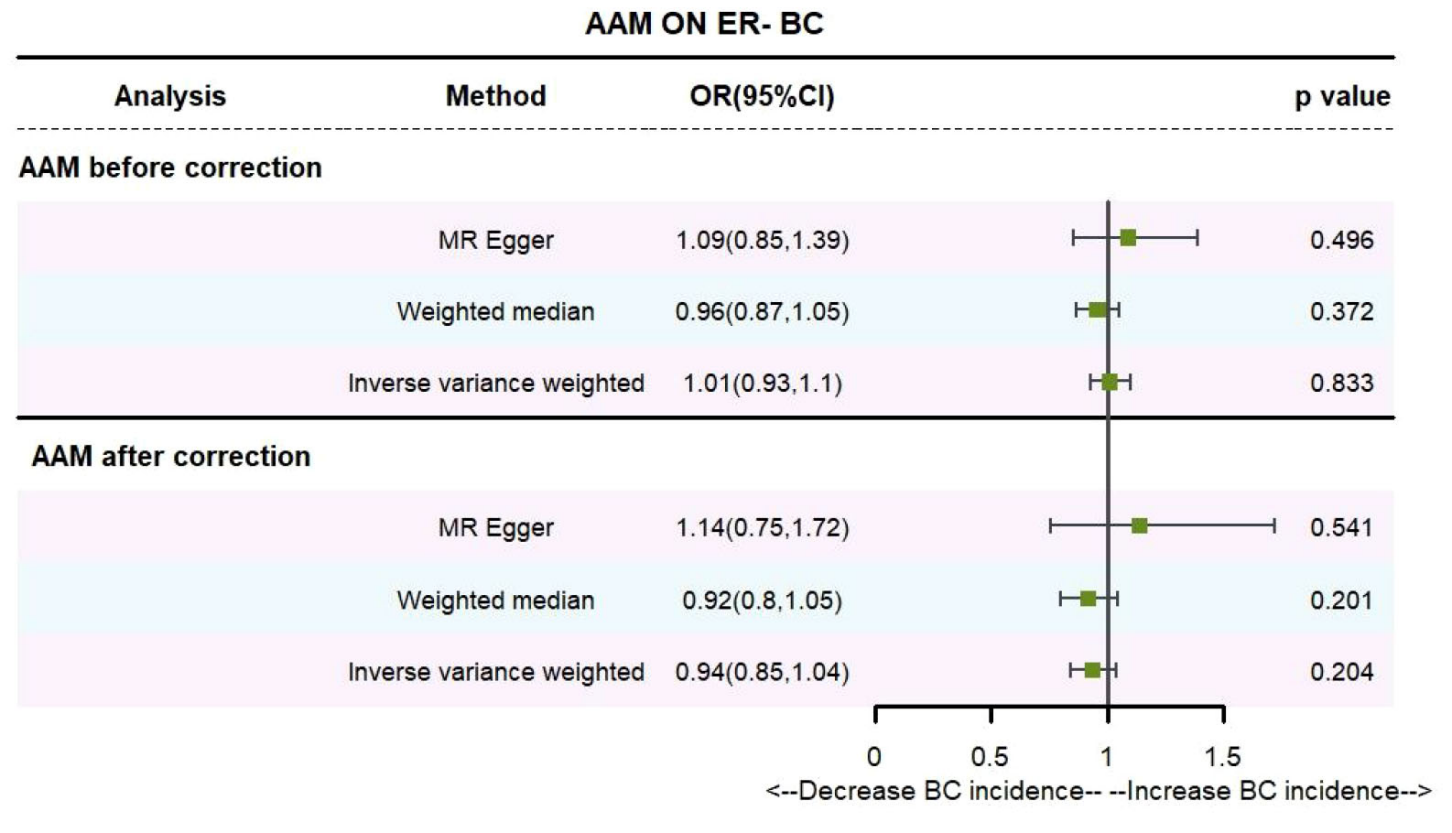
Causality of AAM with ER- BC OR chart for AAM and ER- BC.

Utilizing the IVW method, subsequent to the adjustment for confounders within Phenoscanner, the effect sizes for AAM on ER+ BC and ER- BC were observed to be (OR, 0.895 [95% CI, 0.814–0.983], P=0.021, **Figure 6**) and (OR, 0.936 [95% CI, 0.845–1.037], P=0.204, **Figure 7**), respectively.

Employing the MR-Egger regression approach, the effect size for AAM on ER+ BC was determined to be (OR, 0.724 [95% CI, 0.491–1.068], P=0.111, **Figure 6**), and for AAM on ER- BC, the effect size was (OR, 1.139 [95% CI, 0.754–1.721], P=0.54, **Figure 7**). Furthermore, the weighted median estimation revealed effect sizes of (OR, 0.894 [95% CI, 0.819–0.974], P=0.011, **Figure 6**) for AAM on ER+ BC and (OR, 0.916 [95% CI, 0.800–1.048], P=0.201, **Figure 7**) for AAM on ER- BC.

The collective findings from these analyses suggest that later AAM exerts a significant potential causal influence and confers an reduced risk for the development of Luminal-a/b BC subtypes. Conversely, no discernible association was detected between AAM and the incidence of HER-2 positive BC or Triple-negative BC.

#### 3.2.1 The sensitivity analyses of causality of AAM with Luminal-a/b BC

**Figure 8:**
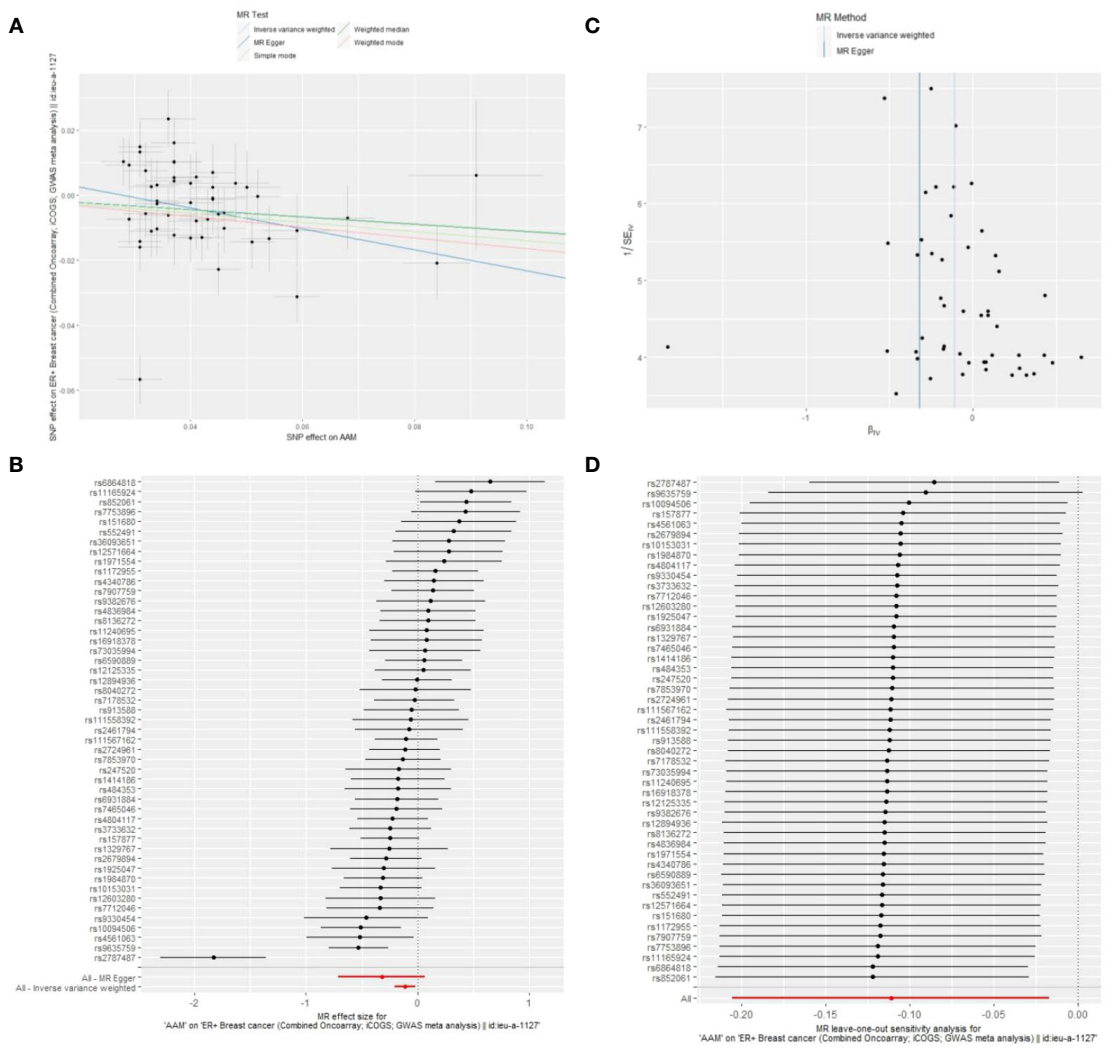
Summary figures of causality of AAM with ER+ BC **(A)** Scatter Plot. **(B)** Forest Plot. **(C)** Funnel Plot. **(D)** Leave-One-Out Plot.

**Figure 9:**
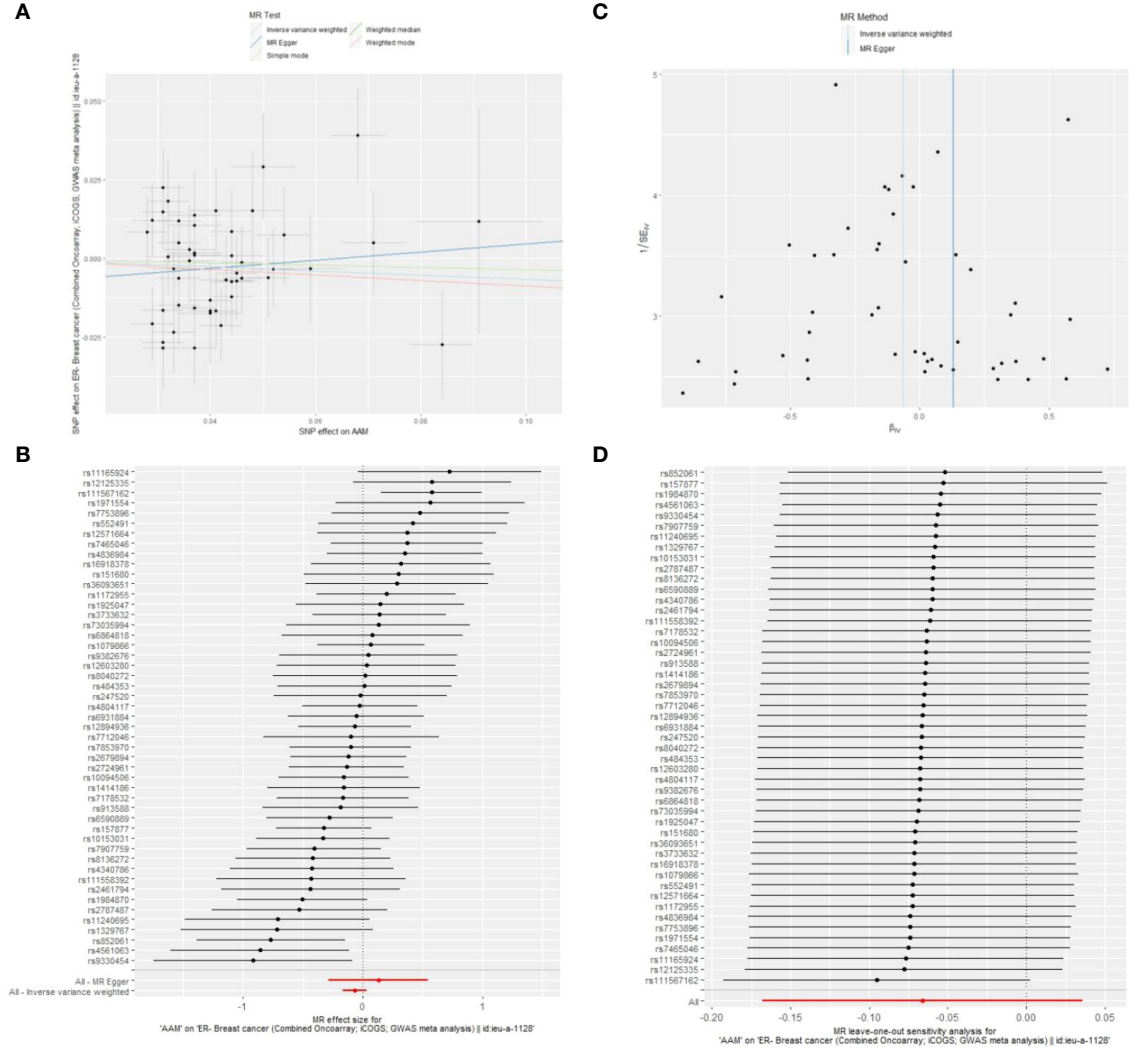
Summary figures of causality of AAM with ER- BC **(A)** Scatter Plot. **(B)** Forest Plot. **(C)** Funnel Plot. **(D)** Leave-One-Out Plot.

Cochran Q test investigated heterogeneity: for AAM on ER+ BC, the result is IVW Q_pval=3.476167e-09, and for ER- BC, the result is IVW Q_pval=0.059. The heterogeneity may be due to the characteristics of different study populations and measurement errors in different studies, which does not affect the reliability of causal inference. Pleiotropy detection: for AAM on ER+ BC, the result is egger_intercept P=0.278, and for ER- BC, the result is egger_intercept P=0.342. MR-PRESSO: for AAM on ER+ BC, the result is P<0.001, and for ER- BC, the result is P=0.041. These findings suggest an absence of heterogeneity and outlier effects. In sensitivity analyses where individual SNP were systematically excluded, no single SNP was found to create a substantial effect on the results, demonstrating the stability. The funnel plot shows that the heterogeneity bias in our study is not statistically significant and is symmetrical in shape (**Figures 8**, **9**) (**Table 2**).

**Figure 8** The scatter plot demonstrates a negative correlation between AAM and BC; Leave-one-out analysis indicates that the comprehensive effect remains unchanged or reverses after the removal of any SNP, suggesting the reliability of the results. This supports the presence of a negative correlation between the AAM and BC; Forest Plot demonstrates a negative correlation between AAM and BC; Funnel Plot exhibits a symmetrical pattern.

#### 3.2.2 The AAM was related to BC in first-degree relatives

**Figure 10:**
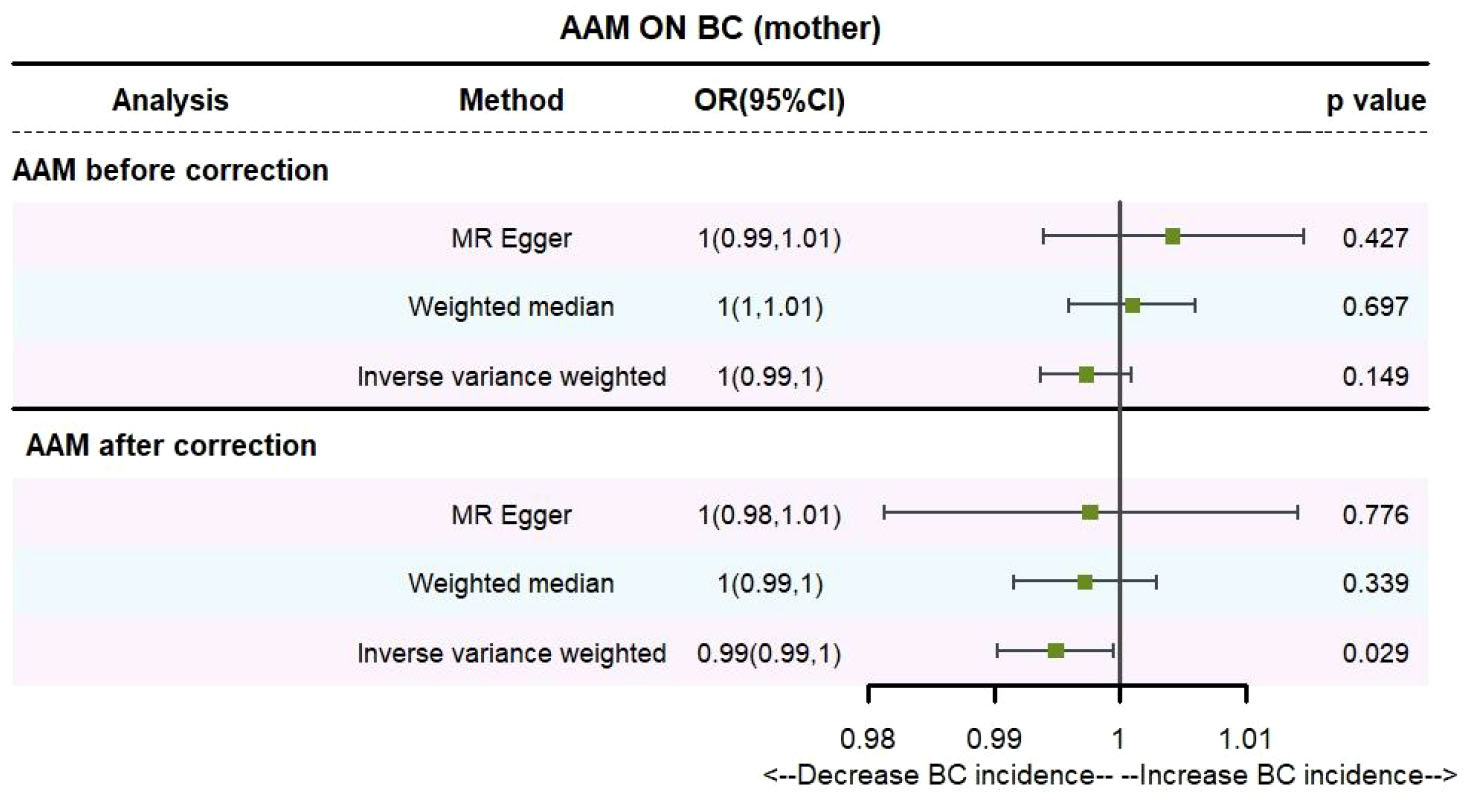
Causality of AAM with maternal BC OR chart for AAM and BC (mother).

**Figure 11:**
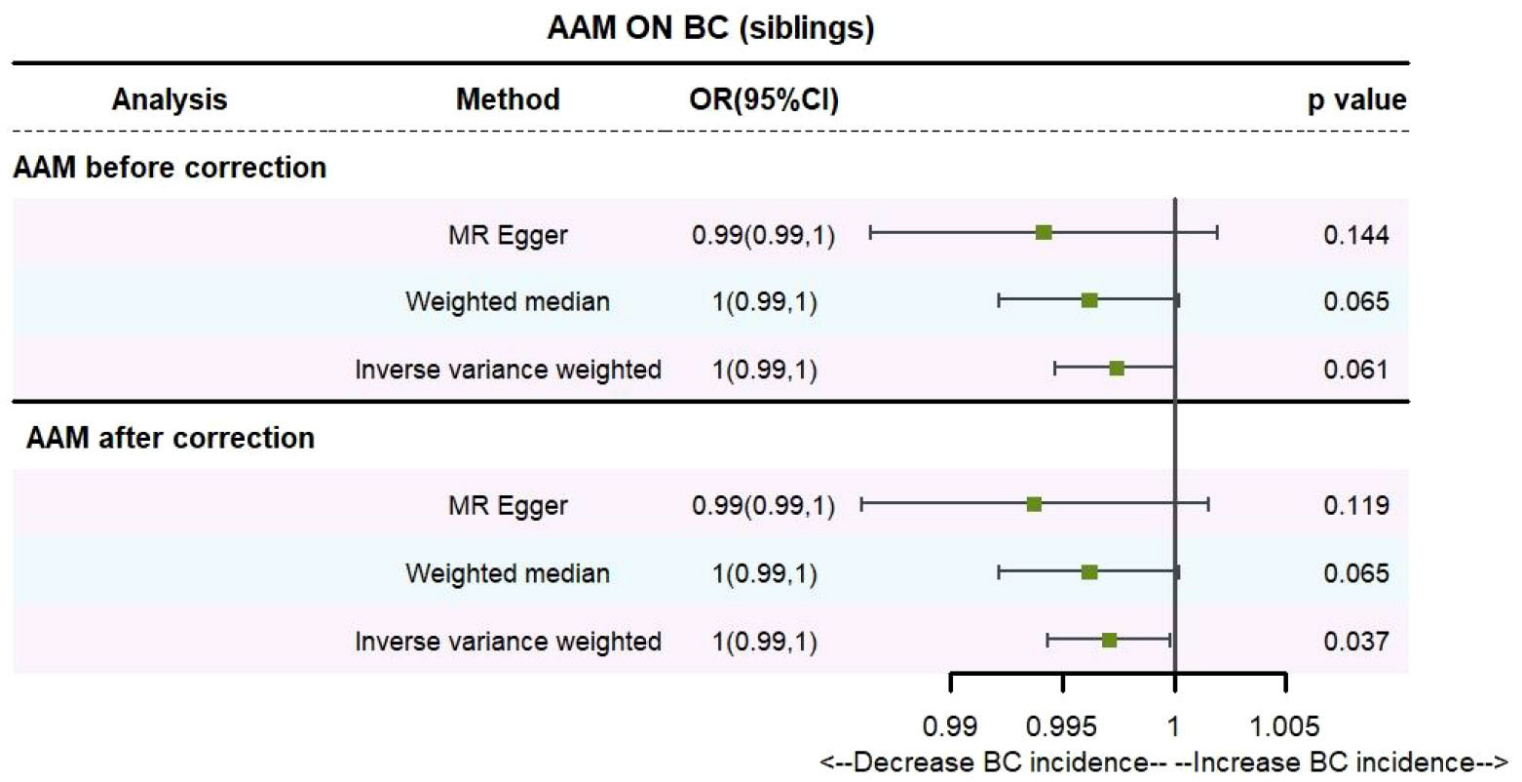
Causality of AAM with sibling BC OR chart for AAM and BC (siblings).

Utilizing IVW method, subsequent to the adjustment for confounding variables within Phenoscanner, the effect sizes for AAM on maternal BC and sibling BC were revised to (OR, 0.995, [95% CI, 0.990–0.999], P=0.029, **Figure 10**) and (OR, 0.997, [95% CI, 0.994–0.999], P=0.037, **Figure 11**), respectively.

Employing the MR-Egger regression analysis, the effect size for AAM on maternal BC was calculated to be (OR, 0.998, [95% CI, 0.981–1.014], P=0.776, **Figure 10**), and for sibling BC, the effect size was (OR, 0.994, [95% CI, 0.986–1.002], P=0.119, **Figure 11**). Furthermore, the weighted median estimation method revealed that the effect sizes for AAM on maternal BC and sibling BC were (OR, 0.997, [95% CI, 0.992–1.003], P=0.339, **Figure 10**) and (OR, 0.996, [95% CI, 0.992–1.000], P=0.065, **Figure 11**), respectively.

Based on our analysis, we found that AAM has a causal effect on the risk of BC in mother and siblings, which provides insights for disease research.

#### 3.2.3 The sensitivity analyses of causality of AAM with BC in first-degree relatives

**Figure 12:**
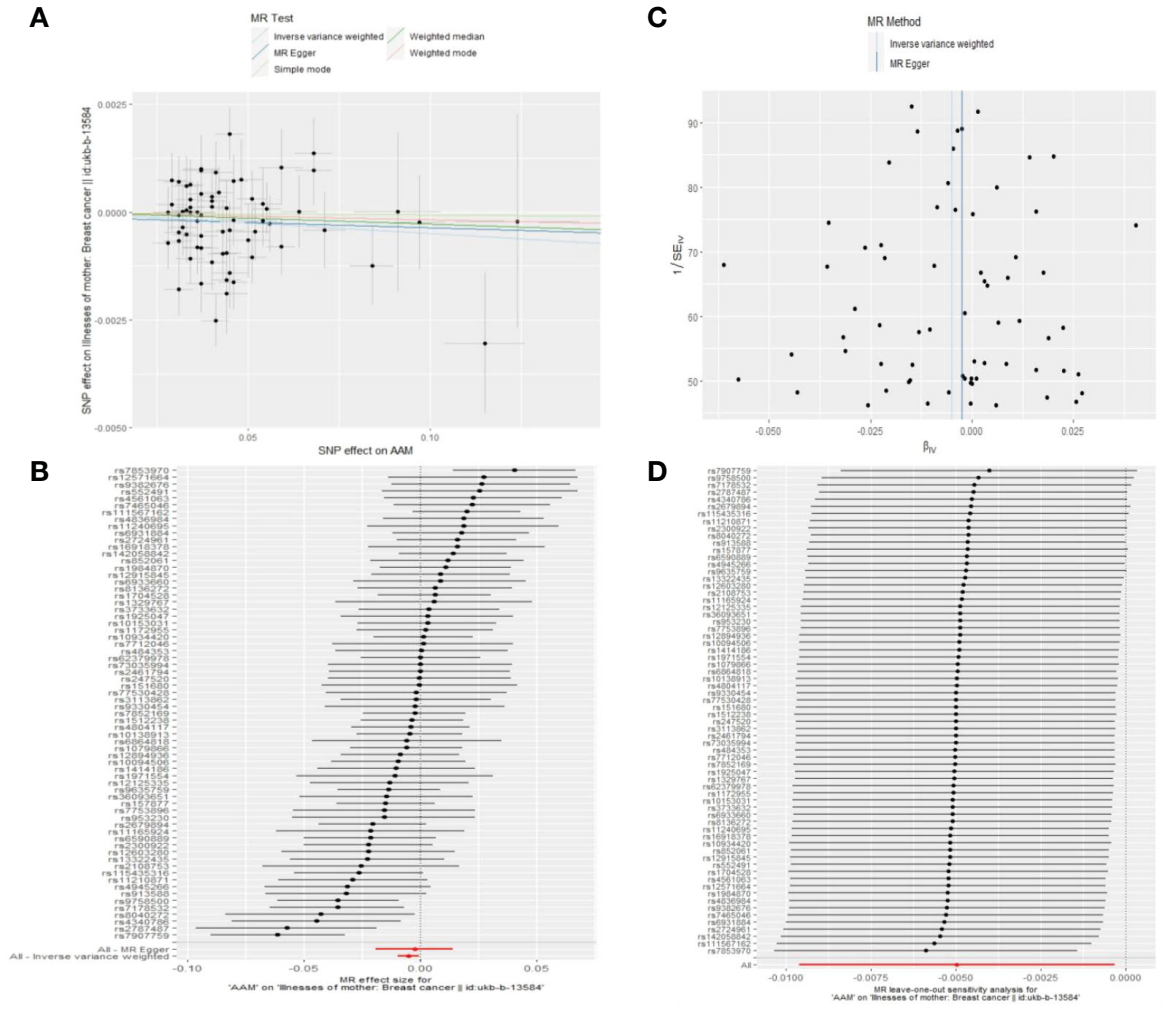
Summary figures of causality of AAM with maternal BC. **(A)** Scatter Plot. **(B)** Forest Plot. **(C)** Funnel Plot. **(D)** Leave-One-Out Plot. The scatter plot demonstrates a negative correlation between AAM and BC. Leave-one-out analysis indicates that the comprehensive effect remains unchanged or reverses after the removal of any SNP, suggesting the reliability of the results. This supports the presence of a negative correlation between the AAM and BC. Forest Plot demonstrates a negative correlation between AAM and BC. Funnel Plot exhibits a symmetrical pattern.

**Figure 13:**
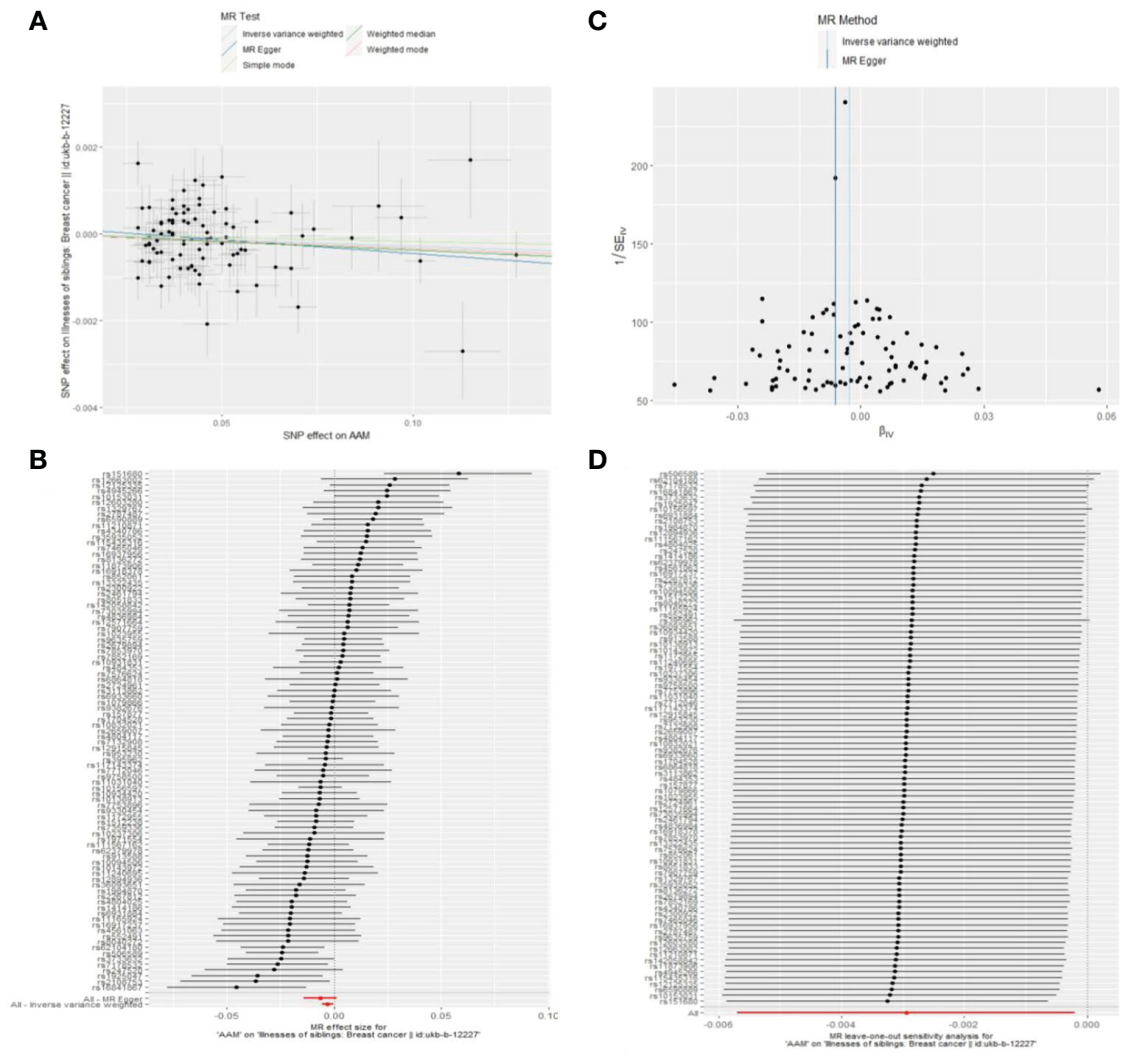
Summary figures of causality of AAM with sibling BC. **(A)** Scatter Plot. **(B)** Forest Plot. **(C)** Funnel Plot. **(D)** Leave-One-Out Plot. The scatter plot demonstrates a negative correlation between AAM and BC. Leave-one-out analysis indicates that the comprehensive effect remains unchanged or reverses after the removal of any SNP, suggesting the reliability of the results. This supports the presence of a negative correlation between the AAM and BC. Forest Plot demonstrates a negative correlation between AAM and BC. Funnel Plot exhibits a symmetrical pattern.

Cochran Q test investigated heterogeneity: for AAM on maternal BC, the result is IVW Q_pval=0.003, and for sibling BC, the result is IVW Q_pval=0.043. Pleiotropy detection: for AAM on maternal BC, the result is egger_intercept P=0.736, and for sibling BC, the result is egger_intercept P=0.375. MR-PRESSO: for AAM on maternal BC, the result is P=0.004, and for sibling BC, the result is egger_intercept P=0.026. In sensitivity analyses where individual SNP were systematically excluded, no single SNP was found to create a substantial effect on the results, demonstrating the stability. The funnel plot shows that the heterogeneity bias in our study is not statistically significant and is symmetrical in shape. (**Figures 12**, **13**) (**Table 2**).

## 4 Discussion

Utilizing two MR approaches, our study substantiated the association between delayed AAM ranging from 15 to 18 years and a diminished risk of BC development, with a notably lower incidence observed in ER+ BC. Moreover, we also found a causal relationship between AAM and the incidence of BC in mother and siblings, which provides certain insights for the study of the disease in a vertical context. Given the pivotal role of the ER as a prognostic marker in BC, a more favorable prognosis is demonstrated in cases of ER+ BC when contrasted with ER- BC (12, 13). Over the past two centuries, AAM in developed countries has remained stable at approximately 13.0 years ± 0.5 years (14). Girls with earlier menarche experience higher levels of estrogen in both early and late life stages (15, 16). An additional putative mechanism posits that an earlier onset of menarche extends the gap between AAM and the first full-term fetation, consequently prolonging the period of estrogen exposure. (17).

Estrogen has been demonstrated to directly arouse the proliferation of BC cells expressing estrogen receptor alpha (ERα). Furthermore, E2 can increase intratumoral vascular density and transform the neoplasm blood-vessel system into a more regular construction, enhancing tumor tolerance, by activating stromal ERα (extra-hematopoietic Tie-2 positive cells), thereby promoting the increase of ERα-negative neoplastic cells (18).Compared to womankind who experienced AAM before thirteen, AAM at or after fifteen years old is associated with a 24% reduction in the risk of ER+ progesterone receptor-positive (PR+) tumors (HR: 0.76 [95% CI: 0.68–0.85]) (19).

Mouse mammary stem cells (MaSCs) exhibit heightened sensitivity to steroid hormone signaling, notwithstanding the lack of ER and PR. Ovariectomy-induced hormonal deprivation significantly impacts the function and gene expression of MaSCs, leading to reduced regenerative capacity and alterations in cell cycle regulation, whereas MaSCs activity increased in mice treated with oestrogen plus progesterone. This demonstrates the roles of both estrogen and progesterone in regulating MaSCs activity. The study also underscores the importance of estrogen stimulation in maintaining normal MaSCs functionality. Ovariectomy in mice results in an increase in the number of cells in the G0/G1 phase and a decrease in the levels of cell cycle regulatory factors Cyclin D1 and Cyclin D2, indicating a reduction in MaSCs’ resulted in a lower incidence of BC. Conversely, an increase in menstrual cycles may lead to an expanded MaSCs population, thereby increasing the individual’s likelihood of developing BC (20).

Another potential mechanism involves long non-coding RNAs (lncRNA). In recent years, it has become increasingly evident that dysregulated lncRNA expression is a hallmark of major pathologies, including cancer (21–24). Among these, GAS5 is considered to have a strong association with BC.

In 1988, during the pursuit of novel tumor suppressor genes, Philipson, L.A.B., et al. successfully isolated GAS5 by utilizing subtractive cDNA cloning techniques (25). GAS5 is a non-protein-coding gene that regulates cell proliferation and is considered an oncogenic suppressor lncRNA in various cancers, including BC (26).

The GAS5 operates as a lure glucocorticoid response element (GRE) through its interaction with the DNA-binding domain of the glucocorticoid receptor (GR), thereby inhibiting the receptor’s transcriptional activity. This interaction inhibits glucocorticoid-mediated transcription of anti-apoptotic genes, thereby increasing the sensitivity of cells to apoptosis (27).

Additionally, GAS5 competitively binds to the endogenous miR-21, targeting phosphatase and tensin homologs (PTEN), leading to increased PTEN levels. This increase in PTEN levels inhibits cancer proliferation. Diminished expression of GAS5 is associated with enhanced cellular proliferation and tumorigenesis. In tumor tissues, GAS5 expression is significantly reduced in comparison to surrounding non-cancerous tissues (28).

It is well-established that mitochondria are pivotal in modulating the cellular apoptotic pathway. Research indicates that the conserved apoptosis family members, BAX (29) and BAK (30), induce cell cytochrome c release by forming voltage-dependent anion channel pores or by permeabilizing the mitochondrial membrane (31, 32). This leads to mitochondrial dysfunction and cell apoptosis (33).Research has confirmed that GAS5 induces mitochondrial membrane potential dysfunction *in vitro*, triggering mitochondria-dependent apoptosis. Furthermore, the expression of key apoptotic regulators such as BAK and BAX has been detected (34).

Participants in experiments with later menarcheal onset exhibited higher levels of GAS5 expression compared to those with earlier menarcheal onset (35). This underscores the significant role of GAS5 in mediating the association between AAM and the pathogenesis of BC.

Our investigation is subject to certain constraints. Primarily, the GWAS data employed in our analysis predominantly originate from European bloodlines, which restricts the extrapolation of our discovery to other racial or ethnic cohorts. Further research with diverse datasets is needed to better understand the association between AAM and BC in different populations. Secondly, the absence of data pertaining to PR and HER-2 precluded a granular examination of the interplay between AAM and various BC phenotypes. Future investigations that integrate these elements may yield a more nuanced comprehension of the nexus between AAM and BC. Our present analysis is circumscribed within the confines of evidence-based medicine, focusing on the scrutiny of clinical statistical data. As for the intricate molecular biological mechanisms that underpin the relationship among precocious AAM and an enhanced BC, further empirical research is keenly awaited.

In summary, given the escalating incidence of BC in recent years, it is anticipated that by 2020, this malignancy will emerge as the predominant cause of cancer-related morbidity among women globally. (36). Following an exhaustive examination of extensive epidemiological evidence via Mendelian randomization, our investigation has innovatively established a causal nexus between delayed AAM and a diminished risk of BC. Additionally, our analysis corroborated the prognostic significance of AAM concerning the occurrence of BC among first-degree relatives. Previous studies have indicated that overweight girls tend to experience menarche at an earlier age compared to girls with a normal BMI (37, 38). Dietary fiber may delay the onset of AAM by influencing estrogen metabolism and reducing the intestinal absorption of estrogen (39, 40). An adverse family environment can also lead to an earlier onset of AAM in girls (41–43). Intense physical exercise can delay the onset of AAM (44). This suggests that a healthy lifestyle and positive family relationships can be considered as early preventive measures against BC. For women who experience early AAM, it is advisable to initiate BC screening earlier and pay close attention to the health of their mother and siblings. Consequently, we advocate for the comprehensive integration of individual menstrual history attributes and hormone receptor status in the development of tailored screening protocols within the ambit of BC prophylaxis and therapeutic modalities. Through the meticulous refinement of BC preventative measures and the enhancement of early detection, our objective is to substantially abate the grave impact of BC on women’s health.

## Data availability statement

The original contributions presented in the study are included in the article/**Supplementary Material**. Further inquiries can be directed to the corresponding author.

## Ethics statement

Ethical approval was not required for the study involving humans in accordance with the local legislation and institutional requirements. Written informed consent to participate in this study was not required from the participants or the participants’ legal guardians/next of kin in accordance with the national legislation and the institutional requirements.

## Author contributions

ZZ: Conceptualization, Data curation, Investigation, Methodology, Software, Validation, Writing – original draft, Writing – review & editing. JZ: Investigation, Methodology, Writing – review & editing. XT: Conceptualization, Methodology, Resources, Supervision, Writing – review & editing.
